# A multi-centered study of *Pneumocystis jirovecii* colonization in patients with respiratory disorders: Is there a colonization trend in the elderly?

**DOI:** 10.18502/cmm.5.3.1742

**Published:** 2019-09

**Authors:** Mahdi Abastabar, Elham Mosayebi, Tahereh Shokohi, Mohammad T. Hedayati, Mohammad R. Jabari Amiri, Zahra Seifi, Iman Haghani, Masoud Aliyali, Sassan Saber, Maryam-Fatemeh Sheikholeslami

**Affiliations:** 1 Invasive Fungi Research Center, Mazandaran University of Medical Sciences, Sari, Iran; 2 Department of Medical Mycology, School of Medicine, Mazandaran University of Medical Sciences, Sari, Iran; 3 Student Research Committee, School of Medicine, Mazandaran University of Medical Sciences, Sari, Iran; 4 Department of Internal Medicine, School of Medicine, Mazandaran University of Medical Sciences, Sari, Iran; 5 Department of Internal Medicine, School of Medicine, Tehran University of Medical Sciences, Tehran, Iran; 6 Department of Molecular Pathology, National Research Institute of Tuberculosis and Lung Disease, Shahid Beheshti University of Medical Sciences, Tehran, Iran; 7 Department of Molecular Biology, Dr. Khosroshahi Pathobiology Laboratory, Tehran, Iran

**Keywords:** Colonization, Immunocompetent, Immunosuppressed, Mitochondrial large subunit, (mtLSU), Pneumocystis jirovecii, Respiratory failures

## Abstract

**Background and Purpose:**

*Pneumocystis jirovecii* colonization plays a key role in the progression of pulmonary infection. However, there are limited data regarding the colonization of these fungi in the patients residing in different regions of Iran. Regarding this, the present study was conducted to evaluate the prevalence of *P. jirovecii* colonization in non-HIV-infected patients with respiratory failure introduced by physicians using nested polymerase chain reaction (PCR).

**Materials and Methods:**

This study was conducted on 136 samples obtained from 136 patients with respiratory disorders referring to different hospitals in the capital and north of Iran during 2013-2015. The samples were collected using bronchoalveolar lavage (BAL; n=121) and sputum induction (n=15). Nested PCR method targeting mtLSU rRNA gene was used for the detection of *P. jirovecii *DNA in the specimens.

**Results:**

The nested PCR analysis resulted in the detection of *P. jirovecii* DNA in 32 (23.5%) patients. The mean age of the participants was 49.04±11.94 years (age range: 14-90 years). The results revealed no correlation between *Pneumocystis* colonization and gender. The studied patients were divided into two groups of immunocompromised and immunocompetent patients. In the regard, 25.4% of the patients with detectable *P. jirovecii* DNA were immunocompromised and had cancer, organ transplantation, asthma, sarcoidosis, dermatomyositis, chronic obstructive pulmonary disease, bronchiectasis, and pulmonary vasculitis. On the other hand, *Pneumocystis *DNA was detected in 21.8% of the immunocompetent patients. Frequencies of *P. jirovecii* DNA detection in the patients with tuberculosis, hydatid cyst, and unknown underlying diseases were obtained as 20.8%, 25%, and 22%, respectively. The prevalence of *Pneumocystis *colonization varied based on age. In this regard, *P. jirovecii* colonization was more prevalent in patients aged above 70 years.

**Conclusion:**

As the findings indicated, non-HIV-infected patients, especially the elderly, had a high prevalence of *P. jirovecii* colonization. Therefore, these patients are probably a potential source of infection for others. Regarding this, it is of paramount importance to adopt monitoring and prophylactic measures to reduce this infection.

## Introduction

*Pneumocystis jirovecii* is an opportunistic fungus, leading to the development of severe interstitial *Pneumocystis* pneumonia (PCP) in immunocompromised individuals, such as HIV-positive patients with CD4 cell counts of ≤ 200 cells/mm^3^. There are asymptomatic or mild pulmonary infections, defined as colonization, in HIV and non-HIV immunocompromised patients [1]. As indicated in the literature, *P. jirovecii *is usually recovered from the respiratory tracts of 20-65% of immunocompetent subjects [1, 2].

The first victims of PCP were malnourished children in the European countries. This infection was also reported in the Iranian orphan infants and children during the Second World War [3]. After 1954, sporadic cases of *Pneumocystis* infection were reported in all countries across the world [4]. With regard to the low sensitivity of the microscopic examination in the diagnosis of this fungal infection and lack of suitable culture media for isolating the associated microorganisms from clinical samples, this disease is likely to have a high incidence. 

There are a number of polymerase chain reaction (PCR)-based assays, such as real-time PCR, nested-PCR, DNA sequencing, restriction fragment length polymorphism, and single-stranded conformation polymorphism, that have been more successful in the detection of these fungi [5-9]. 

Based on the evidence, *P. jirovecii* can colonize in immunosuppressed patients, such as those undergoing systemic corticosteroid therapy or chemotherapy (due to malignancy), as well as HIV-positive patients and cases with collagen vascular disease [3, 10, 11]. In recent decades, the application of highly active antiretroviral therapy and prophylactic treatment against *Pneumocystis* has caused a decrease in the incidence rate of PCP among HIV-positive patients in developed countries. 

However, this infection still has a high incidence rate among immunocompromised patients in developing countries [12]. Despite numerous studies on the colonization of fungus in immunosuppressive individuals in the world, there are limited reports about the prevalence rate of this disease in the Iranian immunosuppressed patients [13-18]. Accordingly, little is known about the prevalence of *Pneumocystis* carriage in non-HIV-infected patients with respiratory failure. With this background in mind, the present study was performed to detect *P. jirovecii* DNA in patients with respiratory disorders in different regions of Iran.

## Materials and Methods

***Samples and patients***

This cross-sectional study was conducted on respiratory specimens obtained from patients with respiratory disorders. The patients were considered as respiratory failure cases with syndromes in which the respiratory system fails in gas exchange functions, namely oxygenation and/or carbon dioxide elimination. They were introduced to the project by the attending physician as a part of the request of the standard medical inquiry for possible *P. jirovecii* DNA colonization between August 2013 and September 2015. The study population was selected from the patients admitted to referral hospitals (affiliated to the Universities of Medical Sciences) for respiratory diseases in different geographical regions of Iran, namely Shariati Hospital (Tehran, Iran), Masih Daneshvari Hospital (Tehran, Iran), Imam Khomeini Hospital (Sari, Iran), Razi Hospital (Ghaemshahr, Iran), and Mycology Laboratory of Toba Polyclinic (Sari, Iran). The samples were collected using bronchoalveolar lavage (BAL; n=121) and sputum induction (n=15). Induced sputum samples were collected by the nurse with an inducer sputum instrument, and a medicine was prescribed which promoted the secretion of the sputum. If the introduced patients suffered from pneumonia, they were excluded from the study. The patients with positive result in nested PCR without any signs or symptoms of pneumonia were considered colonized cases. All of the patients were seronegative for HIV based on enzyme-linked immunosorbent assay. Patients’ demographic and clinical data, including age, gender, clinical manifestation, and predisposing factors, were recorded in a data sheet.

The patients were classified into two groups: 1) the first group included immunocompromised patients who received systemic corticosteroids or were under chemotherapy, 2) the second group included immunocompetent patients who were not under chemotherapy or corticosteroids therapy but suffered from infectious diseases.

***Staining and microscopic test***

All samples were examined microscopically by Gomori Methenamine-Silver and calcofluor white staining for the presence of *P. jirovecii* as described previously [17].

***DNA extraction and polymerase chain reaction***

The viscous respiratory specimens were liquefied with pancreatin solution (0.5%) for 30 min at 30°C, and then concentrated by centrifugation at 3,000 rpm for 5 min. In the next stage, 300 µl of sediment was digested with 20 µl proteinase K (10 µg/µl) at 56°C. DNA was extracted from the digested samples using a commercial kit (QIAamp DNA Mini Kit; Qiagen, Hilden, Germany) following the manufacturer's instructions. 

*Pneumocystis *mitochondrial large subunit (mt-LSU) rRNA gene was amplified using primers pAZ102-H (5′-GTG TAC GTT GCA AAG TAC TC -3′) and pAZ102-E (5′-GAT GGC TGT TTC CAA GCC CA -3′) for primary PCR, as well as pAZ102-X (5′- GTG AAA TAC AAA TCG GAC TAG G -3′) and pAZ102-Y (5′-TCA CTT AAT ATT AAT TGG GGA GC-3′) for nested PCR as described in a similar study [[19]]. The PCR mixture for both rounds of PCR was prepared at a final volume of 25 µL, which contained 2 µL DNA template, 0.5 μl of each primer (25 pmol), 1.25 μl deoxynucleoside triphosphate (5 mM), 0.5 U Taq DNA polymerase, 5 μl 10x PCR buffer, and 15.75 µl distilled water.

Thermal cycling condition included an initial denaturation at 94°C for 2 min, followed by 35 cycles at 94°C for 30 sec, at 55°C for 30 sec, and at 72°C for 1 min, and a final extension at 72°C for 5 min [20]. The nested PCR was performed using a similar thermal cycling profile, except for annealing at 50°C [21]. All amplifications were performed in a Bio-Rad T100 PCR System thermo-cycler (Foster City, CA, USA). The PCR products were visualized on a 1.5% agarose gel with DNA Safe Stain. 

The presence of *Pneumocystis* DNA in the first and second rounds of PCR was considered as PCP. On the other hand, the detection of this fungal DNA in the second PCR round was regarded as colonization or carrier state [[22]]. Sterile distilled water was used to avoid DNA extraction contamination. Furthermore, PCR was performed in a laminar airflow biohazard cabinet using aliquot reagents, positive displacement pipettes, and aerosol resistant tips. In the first round of PCR, sterile distilled water was used as negative control without DNA template. Furthermore, in the second round, the product of the first PCR round was utilized as a negative control.

***Statistical analysis***

The Statistical Package for Social Sciences software (version 22.0, SPSS, Inc., Chicago, IL) was used for statistical analysis. For descriptive analysis, the mean, median, and standard deviation of continuous variables and absolute frequency (percentage) of the categorical variables were calculated. Fisher’s exact and the Chi- tests were performed to compare the groups in terms of the categorical variables. The Q-Q plot graph was drawn based on age group.

***Ethical considerations***

The ethical aspect of the study protocol was approved by the Research Ethics Committee of Mazandaran University of Medical Sciences, Sari, Iran (IR.MAZUMS.REC.94-1552). 

## Results

A total of 136 patients with respiratory disorder had been admitted to different clinical centers under study. The mean age of the patients was 49.04±11.94 years (age range: 14-90 years), and 79% of them were male (n=79). 

**Figure 1:**
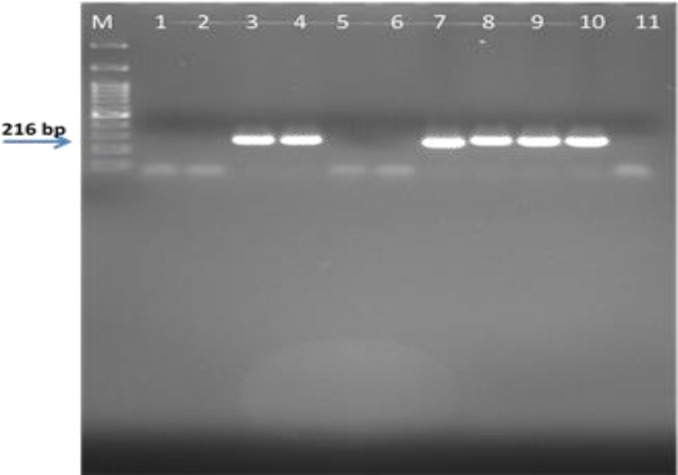
1.5% agarose gel electrophoresis of nested-polymerase chain reaction products of *Pneumocystis jirovecii* in clinical samples; lanes 3-4 and 7-10) positive samples, lanes 1-2 and 5-6) negative samples (The first lane, 100bp molecular ladder.)

In direct microscopic examination, all samples were negative (100%) for the presence of *P. jirovecii*. All respiratory specimens were evaluated for *P. jirovecii* DNA based on the PCR amplification of mtLSU rRNA gene. The two specific primers used in the nested PCR successfully amplified 216 bp fragments of *P. jirovecii* (Figure 1). However, no *Pneumocystis* DNA was detected in the first round of the PCR. Overall, the second round of PCR resulted in the identification of 32 (23.35%) patients with positive *P. jirovecii* DNA, 18 (22.8%) and 14 (10.3%) of whom were male and female, respectively.

Table 1 summarizes the results of PCR based on underlying diseases in the immunocompromised and immunocompetent patients hospitalized. The *P. jirovecii* DNA was detected in 12 (14.6%) immunosuppressed patients suffering from sarcoidosis, pulmonary vasculitis, chronic obstructive pulmonary disease (COPD), dermatomyositis, asthma, and pulmonary failure, as well as organ transplant recipients and those receiving systemic corticosteroids. 

In addition, 1 (25%), 3 (12.5%), and 5 (20.8%) patients with positive *P. jirovecii* DNA had hydatid cysts, different types of cancers (for which they were under chemotherapy), and *Mycobacterium tuberculosis* infection, respectively (Table 1). Out of 59 (43.1%) immunocompromised patients, *P. jirovecii* DNA was detected in 15 (25.4%) cases. Furthermore, the prevalence rate of *P. jirovecii* colonization in immunocompetent patients was 22.1% (17/77). There was no significant difference between the two groups (Table 2). 

**Figure 2:**
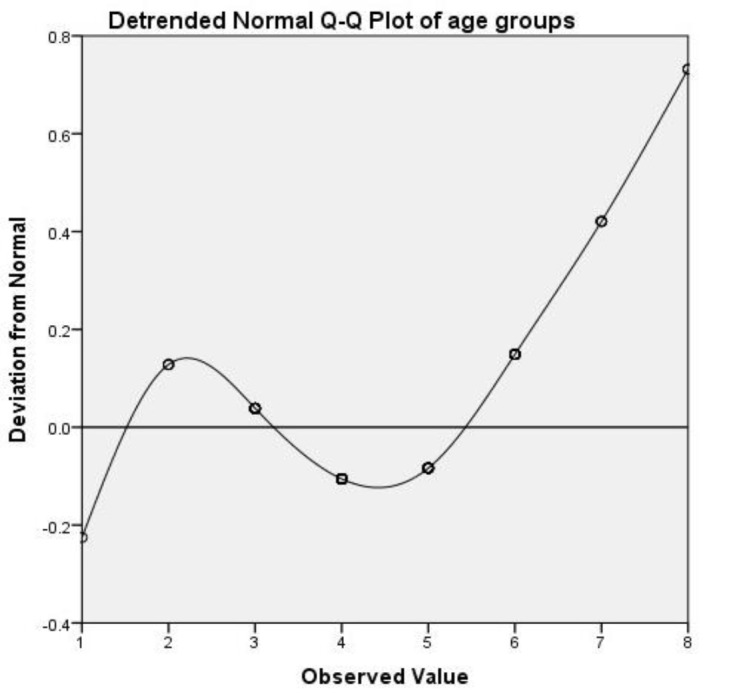
Prevalence of *Pneumocystis jirovecii* colonization based on age group

Table 3 presents the frequency of *P. jirovecii* DNA based on the patients’ age group. The results revealed no significant correlation between *P. jirovecii* DNA colonization and age. However, the prevalence of *P. jirovecii* colonization was found to vary based on age. In this regard, *P. jirovecii* colonization was observed in > 80% and 100% of the patients with the age range of 71-80 and 81-90 years, respectively. Meanwhile, this frequency was obtained as 66.7% among cases in the age group of 21-30 years (Figure 2). Furthermore, there was no significant difference between the males and females regarding the frequency of *P. jirovecii* DNA.

**Table 1:** Results of polymerase chain reaction in the detection of *Pneumocystis jirovecii* DNA based on the underlying disease in the immunocompromised and immunocompetent patients

**Characteristic**	***P. jirovecii*** ** DNA negative** **N (%)**	***P. jirovecii*** ** DNA positive** **N (%)**	**Total patients** **n=137 (100%)**
Immunocompromised patients *			
Cancer	21 (87.5)	3 (12.5)	24(17.5)
Organ transplantation	6 (66.7)	3 (33.3)	9 (6.6)
Chronic obstructive pulmonary disease (COPD)	6 (75)	2 (25)	8 (5.8)
Dermatomyositis	5 (83.3)	1 (16.7)	6 (4.4)
Asthma	3 (60)	2 (40)	5 (3.6)
Sarcoidosis	0	2/2 (100)	2 (1.5)
Pulmonary vasculities and pulmonary failure	3 (75)	1 (25)	4 (2.9)
Bronchiectasis	0	1 (100)	1 (0.7)
Immunocompetent patients			
Tuberculosis	19 (79.2)	5 (20.8)	24(17.5)
Hydatid cysts	3 (75)	1 (25)	4 (2.9)
Unknown respiratory disorders	39 (78)	11 (22)	50 (36.5)

* Due to chemotherapy and immunosuppressive drugs

**Table 2:** Polymerase chain reaction results based on immune status of patients

**Results**		**Immunocompromised**	**Immunocompetent**	**Pearson Chi-Square**
		**Count (%)**	**Count (%)**	
PCR	Positive	15 (25.4)	17 (22.1)	0.687
	Negative	44 (74.6)	60 (77.9)	
Total		59 (100)	77 (100)	

The minimum expected count is 13.88.

**Table 3:** Frequency of *Pneumocystis jirovecii* DNA based on the age groups

**Ratio of ** ***P. jirovecii *** **DNA positive/total patients in that age group**	***P. jirovecii*** ** DNA positive**		**All patients**		**Age group**
**%**	**%**	**No**	**%**	**NO**	
-	-	-	0.7	1	11-20
66.7	6.3	2	2.2	3	21-30
20	15.6	5	18.4	25	31-40
20	34.3	11	40.4	55	41-50
20	18.7	6	21.3	29	51-60
12.5	6.3	2	11.8	16	61-70
80	12.5	4	3.7	5	71-80
100	6.3	2	1.5	2	81-90
23.4	100	32	100	136	Total

## Discussion

In this study, the colonization of *P. jirovecii *was detected in a wide range of non-HIV-infected patients divided into two groups of immunocompromised and immunocompetent patients. *Pneumocystis* colonization is defined as the detection of the organism or its DNA in individuals without any signs or symptoms of acute pneumonia through PCR-based techniques (i.e., nested PCR) [23-25]. A growing attention has been directed toward *Pneumocystis *colonization in the clinical and research domains, which could be due to the important role of this fungus in the development of pulmonary diseases [26]. 

In the current study, although microscopic examination was negative, nested PCR analysis led to the detection of *P. jirovecii* DNA in 32 (23.35%) non-HIV-infected patients. Detection of *Pneumocystis *DNA in 21.8% of the immunocompetent patients is compatible with the results of the recent studies reporting the colonization of this fungus in 20% of healthy cases [27, 28]. Likewise, the *P. jirovecii* colonization rate obtained for immunosuppressed patients in the present study is similar to those reported in other studies (Table 2) [29, 30]. 

Sing et al. [29] using nested PCR method in immunocompetent patients with primary pulmonary disorders obtained a colonization prevalence of 19%, which is close to the rate (17.3%) reported by Khalife et al. [30] in COPD patients. Most of the studies have been focused on the prevalence of *P. jirovecii* in HIV-infected populations [13, 31, 32], while limited reports have investigated the patients suffering from other conditions [2, 15, 33]. Different studies demonstrated that the rate of non-HIV infected populations at the risk of *P. jirovecii* has risen in parallel with the widespread use of corticosteroids, immunosuppressive agents (e.g., TNF-ɑ inhibitors), and organ transplantation [34-36].

Our study showed that the prevalence of *Pneumocystis* colonization varied based on age. In this regard, the colonization rate was high during adolescence, underwent a decline in middle age, and again increased in old age. Accordingly, *Pneumocystis* colonization was observed in 80-100% of the adult patients (age range>70 years; Figure 2). In another study, it was demonstrated that non-immunosuppressed older adults were frequently colonized with *Pneumocystis. *As a result, they suggested that older patients may play the role of a reservoir, thereby increasing the transmission of *P. jirovecii* to immunosuppressed susceptible individuals [37]. In the same vein, our findings suggested that the elderly patients with different kinds of pulmonary disorders may be a source of *Pneumocystis*, through whom this infection is transmitted to high-risk individuals.

In the present study, the results of the Fisher’s exact test and Chi-square test demonstrated no significant difference between the immunocompromised and immunocompetent patients considering the prevalence of pulmonary *P. jirovecii* colonization (25.4% vs. 21.8%; P=0.685) (Table 2). In line with the findings obtained by Nevez et al., our results indicated that the colonized immunocompetent patients may serve as a reservoir for *P. jirovecii* [38]. 

Our results also revealed no significant correlation between the prevalence of *P. jirovecii* colonization and the underlying disease. However, the immunocom-promised patients using systemic corticosteroids had a high prevalence of *Pneumocystis* colonization (Table 1). There are a number of studies reporting a relationship between *Pneumocystis* colonization and the underlying diseases (e.g., acute renal transplant rejection) using nested PCR [38, 39]. 

In the current study, *P. jirovecii* DNA was detected in 100%, 40%, 33.3%, 25%, 16.7%, and 12.5% of the patients with sarcoidosis/bronchiectasis, asthma, organ transplantation, COPD/pulmonary vasculitis, dermatomyositis, and cancer, respectively. Our data cannot be generalized because the employment of a small sample size. Consequently, it is recommended to design a multicenter study with a larger sample size to find any probable correlation between the colonization of infection and underlying diseases and investigate its role in pulmonary diseases.

In the present study, *P. jirovecii* colonization was relatively common among patients with tuberculosis (TB; 20.8%). The results of this study confirm our previous research findings showing a high rate of PCP and TB co-infection among the Iranian HIV-infected patients [40]. Similarly, Aboualigalehdari et al. reported a high colonization prevalence (27.0%) in TB-positive patients [15]. Since TB is an endemic disease in Iran, the colonization of these opportunistic agents in these patients has turned them into a reservoir of infection. 

Notably, the lower prevalence of PCP in the HIV-positive patients can concurrently result from the implementation of anti-TB treatment and prophylactic measures against pneumonia. Based on the World Health Organization data, Iran is listed as an endemic area of TB. Accordingly, the Ministry of Health and Medical Education of Iran has adopted anti-TB care programs for all HIV patients [41]. There is a logical relationship between TB and PCP because TB as a debilitating disease can increase *P. jirovecii* colonization and lead to the progression of this condition from a latent stage to an active disease. 

Although the number of our samples was low, *Pneumocystis* DNA was detected in 2 (25%) patients with COPD, which was indicative of a high rate of colonization in this group of patients. Previous studies showed a significant relationship between COPD and colonization of *Pneumocystis* [29-31, 42]. The adoption of a larger sample size could result in the achievement of a more valid estimation of *Pneumocystis* colonization rate in COPD patients. Our results are in concordance with those of other studies detecting a high prevalence of *P. jirovecii* colonization in patients with organ transplantation [33]. 

Based on the literature, among different non-HIV-positive immunocompromised patients, diabetic patients had the highest frequency of *Pneumocystis* colonization [14]. In the previous report, the patients with autoimmune diseases, such as systemic lupus erythematosus and dermatomyositis, were reported to have a high PCP mortality rate [43, 44]. In the current study, one patient with dermatomyositis (16.7%) who received systemic corticosteroids was colonized with *P. jirovecii.* This could suggest the role of *Pneumocystis* colonization in pulmonary diseases among the immunocompromised patients using systemic corticosteroids. 

One of the limitations of the present study is the low number of patients in some groups with specific risk factors in spite of the relatively large cohort of patients with respiratory disorders. We think that the quantitative real-time PCR can be more helpful in the differentiation of colonization from active infection. Therefore, it is recommended to design a multicenter study to find the probable relationships between *Pneumocystis* colonization and underlying diseased in different groups of non-HIV-positive patients.

## Conclusion

The findings of the present study demonstrated a probably high prevalence of *P. jirovecii* colonization in immunocompetent patients with respiratory disorders. Furthermore, the prevalence of *Pneumocystis *colonization was found to vary based on age. Given the high prevalence of *Pneumocystis *colonization in the elderly immunocompetent patients, they can be considered probable reservoirs for this infection increasing the probability of infection transmission to the susceptible individuals in the society. In the present study, the nested PCR was capable of detecting small amounts of *Pneumocystis* DNA, thereby facilitating the identification of the colonized patients. With regard to the findings of this study, it is mandatory to adopt preventive and monitoring measures to prevent from the transmission of this infection.
